# 

**Published:** 1896-08-08

**Authors:** 


					THE HOSPITAL. ~j ' / ABSOLUTELY PURE, therefore BEST, August 8th, 189
CADBURY'S ^ CADBURY'S
cocoa m anil Im cocoa
" The standard of highest purity at preseni 11^ ALKALIES USED,
attainable."?Lancet.    #
l"'"WrW1CH HOSPITAL
i-ornTi m1TTATJmii??'n
LAs'cotr Western ItiFiRHARf
No. 515. Yol. XX.
Saturday.
August 8th, 1896.
WITH EXTRA NURSING SUFHLhMtNI. *r?AV-0 tn"RY*v/x<-
n> ? x J i ii n 1 D 4. nm *t r Per Annnm. 10s 6d., post free.
[Registered at the General Post Office as a Newspaper for Monthly Parts, 6d.; post free 8d
transmission in the United Kingdom and Abroad.] Dittoper Annnm, 8s. 6d., post free!

				

